# Analysis of the auditory processing skills in 1,012 children aged 6–9 confirms the adequacy of APD testing in 6-year-olds

**DOI:** 10.1371/journal.pone.0272723

**Published:** 2022-08-18

**Authors:** Anna Guzek, Katarzyna Iwanicka-Pronicka

**Affiliations:** 1 The Outpatient of Speech Therapy, The Children’s Memorial Health Institute, Warsaw, Poland; 2 Department of Audiology and Phoniatrics, The Children’s Memorial Health Institute, Warsaw, Poland; 3 Department of Medical Genetics, The Children’s Memorial Health Institute, Warsaw, Poland; All India Institute of Speech and Hearing, INDIA

## Abstract

The aim of the study was to assess the validity of the use of the battery of tests assessing higher auditory functions in the diagnostic process of APD in 6-year-old children. The study involved 1,012 Polish-speaking children aged 6 to 9 years with normal hearing sensitivity. The evaluation of auditory functions was performed using the ATS Neuroflow test battery comprising: Adaptive Speech in Noise test (ASPN-S), Dichotic Digits Test (DDT) and Frequency Pattern Test (FPT). Two groups were distinguished: the group”S” (Study) containing 880 participants with APD (participants who obtained abnormal results in at least two tests) and the group”C” (Control) including 132 participants without APD. The results obtained by 6-year-old children in behavioral tests present a similar disorder’s profile to those of older children in terms of the prevalence of specific deficits and their severity. Performance in the APD tests of healthy 6-year-old children is higher than 9-year-old children with APD, despite the process of physiological development of hearing functions in older children. The test assessing understanding speech in noise was the most frequently impaired among all examined, while the dichotic listening with distracted attention was the least frequently impaired function. The deficit found in DDT was opposite between patients with APD and healthy children, we called the detected phenomenon the reversed lateralization pattern. The use of DDT, FPT and ASPN-S tests to evaluate higher auditory functions in the process of diagnosing APD in 6-year-old children is justified by the lack of discrepancy in the disorder profile of 6-year-old children in comparison with older children, both in the healthy population, and in children with impaired auditory function development. Early diagnosis can be beneficial for accurate programming of therapeutic goals.

## Introduction

Auditory Processing Disorder (APD), currently classified in ICD-10 (International Statistical Classification of Diseases) as H93.25, is defined as a deficiency of auditory stimuli processing in the central part of the auditory system, with the simultaneous normal function of peripheral auditory structures. Importantly, the deficiency involves both verbal and nonverbal stimuli [1]. APD prevents the full use of auditory information, which has a direct impact on a person’s daily life. The diagnostic testing of auditory functions is based on tests which evaluate sound localization and lateralization, auditory discrimination, temporal auditory processing, sound pattern recognition, dichotic listening and the ability to understand distorted speech or speech in the presence of background noise [2]. According to the guidelines of the American Speech-Language-Hearing Association (ASHA), APD is diagnosed in a patient who obtained abnormal results in at least two tests which evaluate auditory functions (an abnormal result means a value below the mean, minus two standard deviations) [2]. APD can manifest at any age. The British Society of Audiology (BSA) distinguished three subtypes of APD [1]. The presented study involved children with developmental APD (diagnosed in children with normal hearing sensitivity).

When difficulties in processing auditory information arise during early childhood, their consequences become particularly important for linguistic, social and emotional development. They can also determine the child’s future educational success [3]. Developmental disorders which are undetected in early childhood and thus untreated may sometimes persist until adulthood [4]. Although the discussion and doubts concerning early therapeutic intervention in children younger than 7 years old, based on the assessment of clinical symptoms, have become increasingly less frequent in the audiological and speech therapy community, the diagnosis of an auditory processing disorder in such children is still controversial, especially if it is based on the analysis of results of behavioral tests assessing higher auditory function.

### Diagnostic APD testing in children aged under 7

Audiological associations worldwide indicate diagnostic paths, however they fail to clearly define the lower age limits for APD diagnosis [2]. BSA [1] raises the issue of the lack of diagnostic testing and therapy in children aged under 7 who exhibit difficulties in auditory perception. At the same time, BSA emphasizes the importance of widely available diagnostic testing and the effectiveness of early therapy in children with impaired hearing. On the other hand, the American Academy of Audiology (AAA) [5] recommends cautious interpretation of the results of psychoacoustic behavioral tests evaluating auditory functions in children aged under 7 or even 8, which is related to the auditory pathway immaturity, insufficient level of cognitive and language development and insufficient attention span. Large intra- and interindividual spread of results reduces the diagnostic value of tests [6].

The need for auditory function testing in children aged under 6 is mentioned by Jerger and Musiek [7] and Munguia [8], who primarily indicate the usefulness of questionnaires assessing clinical symptoms in this age group. On the other hand, a study by Yathiraj and Vanaja [9] describing the assessment of higher auditory functions in 280 healthy children without APD, showed that the testing can be performed even in 6-year-olds. In this study, the authors used, among others: Speech-in-Noise in Indian-English test and Dichotic consonant-vowel test. BSA [1] points out that in addition to standardized tests evaluating auditory perception (including at least one nonverbal test), questionnaires assessing clinical symptoms should be used for diagnosing APD. For example, the Scale of Auditory Behaviors (SAB) is applied in Poland. This test, however, does not sufficiently recognize the specifics of symptoms in younger children who have not started their school education yet [10]. For this reason, the aforementioned scale was not used in the present study. It would be helpful to create an APD risk rating scale that includes clinical symptoms already observable in preschool children (S1 Table) along with a list of APD risk factors (S2 Table), to be used in the diagnosis of APD in younger children [11]. In addition to the treatment of the underlying condition, children who are identified as being at risk of inconsistent development of auditory functions should be examined for auditory behavior and language development in the youngest possible age. Most of the tests used worldwide to evaluate higher auditory functions enable the diagnosis of auditory processing disorder at the age of 7 and older, due to the age standards adopted in the psychoacoustic tests. However, difficulties resulting from underdeveloped auditory perception can be observed at a much younger age–even in children under 3 [12].

Some batteries of tests assessing higher auditory functions using psychoacoustic behavioral tests for children under the age of 7 have been recently elaborated [13–15]. However, these studies, conducted on Portuguese and Danish children, were based on small age groups, counting only 30 children. The test developed for assessing the understanding of speech in noise is the Polish Pediatric Matrix Sentence Test (PPMST) designed for children aged 3 years and more [16]. Its advantage is the optional *point a picture* procedure, which enables administration of the test to children with verbal communication difficulties. On the other hand, the ATS Neuroflow test battery (available at https://www.apd-medical.pl/pl/) includes three standardized tests designed for children aged 5 years and older: Auditory Response Test (ART), Visual Response Test (VRT) and Adaptive Speech in Noise test for words (ASPN-S) requiring repetition of the words heard. Broader diagnostic testing of higher auditory functions is possible in children aged 6 and older when standardized results are available for the following tests: Adaptive Speech in Noise Test for sentences (ASPN-Z), Dichotic Digit Test (DDT) and Frequency Pattern Test (FPT) [17, 18].

This study is aimed to:

- assess the adequacy of the use of the psychoacoustic behavioral tests in evaluation of higher auditory functions in the diagnostic process of APD in 6-year-old children,
- analyze auditory function deficits in 6-year-old children and compare the disorder profile with the results of 7-, 8- and 9-year-old children.

## Material and methods

### Material

The study included a group of 1,012 Polish-speaking children aged 6–9 with normal hearing sensitivity. The participants were recruited from patients of the Department of Audiology and Phoniatrics of the Children’s Memorial Health Institute (CMHI) in Warsaw, Poland, who were diagnosed with APD and healthy students from three Warsaw primary schools. The exclusion criteria included: hearing loss, intellectual disability (based on the parental reports), psychiatric disorders, neurological diseases (i.e. epilepsy) and hereditary syndromes with a known genetic background. Two groups were selected based on the results of the battery of behavioral tests assessing higher auditory functions:

- a group”S” included 880 participants diagnosed with APD based on the audiological testing according to the criteria of ASHA (individuals with at least two tests with abnormal results on the APD test battery),
- a group”C” counted 132 participants in whom APD was excluded. These children obtained normal results in all APD tests or only one test with abnormal result.

Table 1 shows size and percentage of sex distribution across both study and control group, by age group.

**Table 1:** Size and percentage of sex distribution in study and control groups, by age group.

Groups	Group”S”			Group”C”		
Age (years)	Females	Males	Total	Females	Males	Total
**6**	71 (33%)	143 (67%)	214 (24%)	16 (42%)	22 (58%)	38 (29%)
**7**	95 (34%)	185 (66%)	280 (32%)	12 (32%)	26 (68%)	38 (29%)
**8**	69 (31%)	155 (69%)	224 (26%)	7 (27%)	19 (73%)	26 (19%)
**9**	56 (35%)	106 (65%)	162 (18%)	13 (43%)	17 (57%)	30 (23%)
**Total**	291 (33%)	589 (67%)	880	48 (36%)	84 (64%)	132

In the group "S" two-thirds were boys. This reflects the higher APD prevalence in boys and is consistent with the previous epidemiological studies [19]. The group”C” had a similar sex distribution, i.e. two-thirds of the group were boys. Due to the lack of reports regarding gender-specific differences in terms of auditory function development, no analyses from such a perspective were conducted in the present study [6].

Then, based on the declarations of parents, the percentage distribution of the right hand dominance in children in each group was analyzed taking into account age of particular children. The results are shown in Table 2. The percentage of children identified as left-handed by their parents was the same in both groups– 7%. The laterality was not analyzed due to the declarative character of the collected data and the lack of the results of the laterality index test among our examined.

**Table 2:** Percentage left-handedness distribution in both the study group and the control group, by age.

Groups	Group”S”		Group”C”	
Age (years)	Females	Males	Females	Males
**6**	2 (3%)	12 (8%)	0	1 (5%)
**7**	8 (8%)	10 (5%)	3 (25%)	2 (8%)
**8**	2 (3%)	14 (9%)	0	3 (16%)
**9**	4 (7%)	13 (12%)	0	0
**Total**	16 (2%)	49 (8%)	3 (6%)	6 (7%)
	65 (7%)		9 (7%)	

### Methods

ENT consultation was conducted for all participants. Pure Tone Audiometry (PTA), speech audiometry (AC40, Interacoustics, Denmark with TDH39 headphones), tympanometry with acoustic stapedius reflex (ASR) and distortion product otoacoustic emission (DPOAE) (Titan, Interacoustics, Denmark) tests were registered to exclude the diagnosis of peripheral hearing loss according to already published data [13]. In children qualified for testing, correct hearing thresholds within 20 dB was ensured, within the 125 Hz-8 kHz frequency range.

Next, we evaluated the higher auditory functions using a battery of tests available online on the ATS Neuroflow platform. Each participant was examined by APD-certified Neuroflow Provider in an acoustically treated room (the same place for each participant). The tests were administered with an audiometer AC40 (Interacoustics, Denmark) through the TDH39 headphones (Interacoustics, Denmark) and the presentation level was 60 dB, for both ears. The entire procedure lasted 20 minutes for each participant. Each test was commenced after previous confirmation that the examined participant understood the task. In all participants the tests were performed in the following order: 1. Adaptive Speech in Noise test for words (ASPN-S); 2. Dichotic Digit Test (DDT); 3. Frequency Pattern Test (FPT). The obtained results were standardized for the age norms for 6-, 7-, 8- and 9-year old children, respectively S5 Table [17, 18].

#### Test procedure for assessing higher auditory functions

The following tests were used to assess higher auditory functions, in the order given below:

1. ASPN-S test: a test assessing understanding of speech in noise. It involves presenting monosyllabic words in the presence of noise (the presented study used a *multitalker babble*). Monosyllabic words were presented binaurally with variable signal intensity, together with *multitalker babble* which was presented with a constant intensity of 60 dB. The task of the listener was to repeat the heard word. The correct answer was considered to be a precise repetition of a word, while the incorrect—no repetition or inaccurate repetition (e.g. saying a different word). The first two words were given with a large difference between the intensity of the signal and the noise (14 dB), which allowed the subject to give a certain answer. Each subsequent word was delivered 4 dB lower until the listener answered incorrectly. After the incorrect response, the intensity of the”word” stimulus was increased by 4 dB and the stimulus was continued until the correct response was obtained. After the correct response was obtained, the intensity of the stimulus was decreased by 2 dB. The changes in intensity were repeated 5 times. The mean SNR (signal-to-noise ratio) threshold was determined based on the 4 lowest values at which the participant gave the correct answer [17].
2. DDT: a test of dichotic listening with distracted attention. It involves presenting 20 sequences of two different pairs of numbers (1 to 10) to the left and right ear at the same time. The participant’s task is to repeat all four numbers heard in a sequence, the order of the numbers is not relevant. The result is presented as a percentage of correctly recognized sequences for each ear, respectively. This test assesses the maturity of the auditory system in its central part and enables identification of the cerebral hemisphere dominance in terms of verbal stimuli.
3. FPT: a test checking the frequency pattern of sequences. It involves determination of the sequence of the sounds heard. Twenty sequences of three-element tones, of two frequencies (high tone: 1020 Hz, low tone: 880 Hz), are presented. The participant’s task was to recognize and name the heard sounds in the presented order. When the sounds of high, low and high pitch were presented, the child was expected to answer: high, low, high. The result of the test was the percentage of correctly recognized sequences. FPT can be used for assessing the ability to discriminate sound frequencies, level of auditory short-term memory and level of functioning of the right cerebral hemisphere.

The Bioethics Committee of the CMHI approved the study, all data was analyzed anonymously. The study was conducted according to the principles of the Helsinki Convention. The informed consent was given by the parents or legal guardians of our participants. The obtained results were analyzed with MS EXCEL and Statistica software for Windows. Non-parametric tests were used for the analyses, because most of the variables analyzed differed from the normal distribution (Kolmogorov-Smirnov, Lilliefors tests), the results are included in the S3 Table. The ANOVA-Kruskal-Wallis test, the non-parametric equivalent of the analysis of variance and the post-hoc test for the analysis of multiple comparisons of mean rank for all samples were used to compare the variables between the groups. The value of statistical significance was p<0.05.

## Results

The way of presentation of the results is aimed at illustrating the similarities in the profile of the auditory functions of 6-year-old children and older children, which allows for the justified use of the tests in the diagnosis of APD in 6-year-olds. First, we showed the results obtained in the three auditory processing assessment tests (DDT, FPT and ASPN-S) in both groups. Then, we focused on the overall analysis. We compared the number of tests with abnormal results and analyzed the character of the deficit in each age group. Next, looking for similarities in the auditory function profile, we conducted a more detailed analysis for the impact of DDT test results on abnormal results in ASPN-S test and FPT tests in both groups. Finally, we determined the dynamics of the development of individual auditory functions and whether the depth of deficits in auditory functions assessed with individual tests depends on age.

We analyzed the results of tests [17, 18] assessing higher auditory functions (S4 Table) in reference to the age norms for 6-, 7-, 8- and 9-year old children presented as a percentage standard (S4 Table), which allowed for the comparison of the results between different age groups. In the study, we assumed the results as normative when the obtained values corresponded to the standard age values for each test of the battery applied. The reference value for each tests and each age group along with the interpretation of the results are provided in the Supplement, in S5 Table.

### 1. Comparison of the results of auditory function tests among study groups

**Fig 1:**
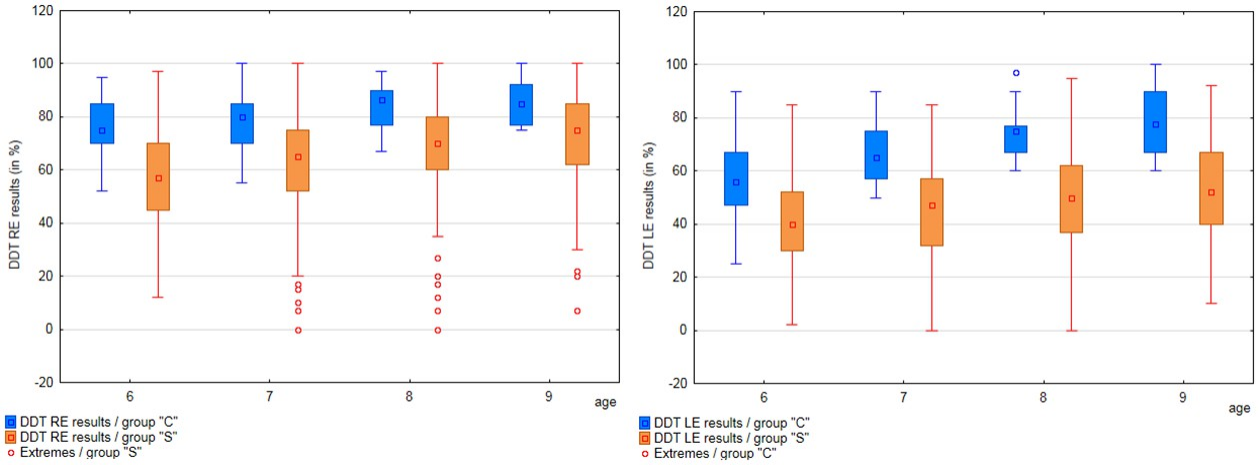
Comparison of DDT RE and LE test results, among age groups, in groups”C” and”S”.

**Fig 2:**
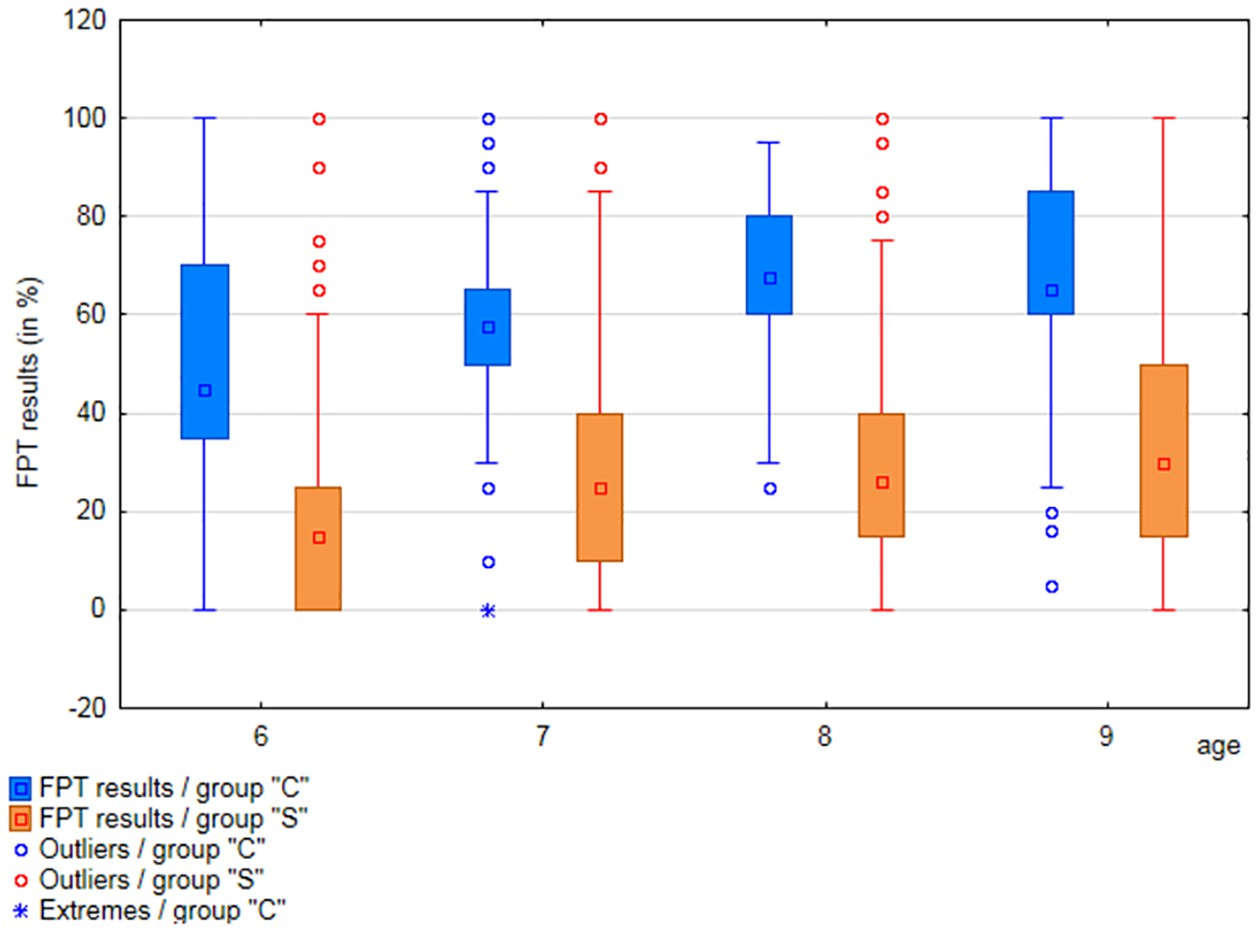
Comparison of the FPT test results, among age groups, in groups”C” and”S”.

**Fig 3:**
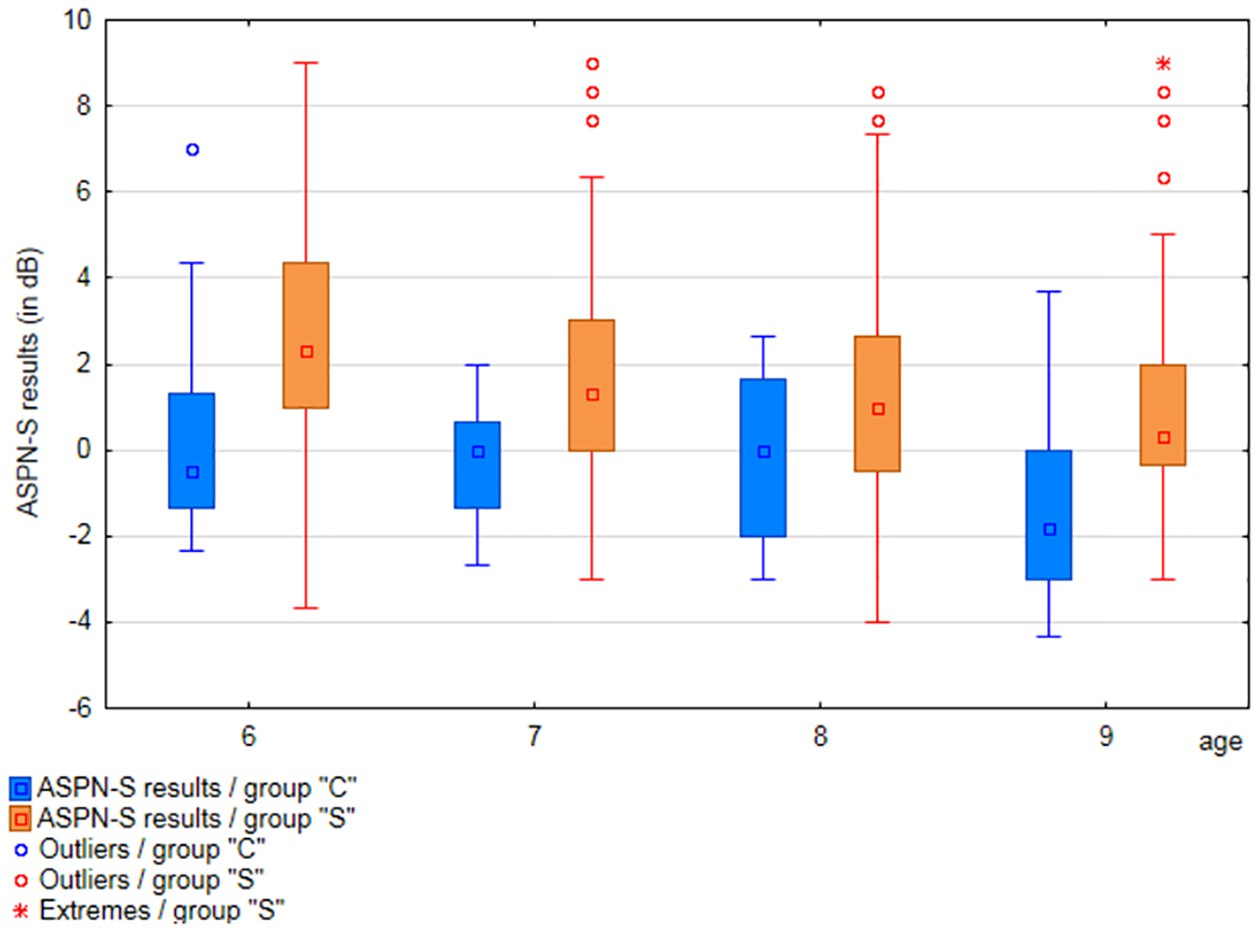
Comparison of the ASPN-S test results, among age groups, in groups”C” and”S”.

Results obtained by participants from each age range in particular APD tests were analyzed (separately for groups”S” and”C”). Results are shown in Figs 1 to 3 and S6.1–S6.3 Table.

The mean values in the DDT test for the right ear (S6.1 Table) in the”S” group ranged from 57.36% (6 years) to 72.12% (9 years), while the standard deviation was from 17.25% to 16.04%. In group”C” the mean values in the DDT RE test ranged from 76.21% (6 years) to 85.63% (9 years), and the standard deviation from 9.48% to 8.24%. In the DDT LE test (S6.1 Table) in the”S” group the mean values ranged from 40.37% (6 years) to 53.37% (9 years), and the standard deviation from 16.37% to 18.69%. The mean values in the DDT RE test in group”C” ranged from 57.66% (6 years) to 78.07% (9 years), and the standard deviation ranged from 16.11% to 12.14%. The participants obtained higher results in the right ear than in the left ear in both groups, in all age groups.

The mean values of the FPT test (S6.2 Table) in the”S” group ranged from 18.2% (6 years) to 33.08% (9 years), while the standard deviation ranged from 21% to 22.9%. In group”C” the mean values in the FPT test ranged from 50.66% (6 years) to 64.03% (9 years), and the standard deviation ranged from 24.64% to 25.71%.

The mean values in the ASPN-S test (S6.3 Table) in the”S” group ranged from SNR 2.92 dB (6 years) to SNR 0.84 dB (9 years), while the standard deviation was from SNR 2.6 dB to 2.1 dB SNR. In the”C” group, the mean values in the ASPN-S test ranged from SNR 0.15 dB (6 years) to SNR -1.23 dB (9 years), and the standard deviation was from SNR 2.07 dB to SNR 1.8 dB.

### 2. Variability of the APD test results profile in both groups

#### 2.1. Quantitative analysis: The number of tests with abnormal results

**Fig 4:**
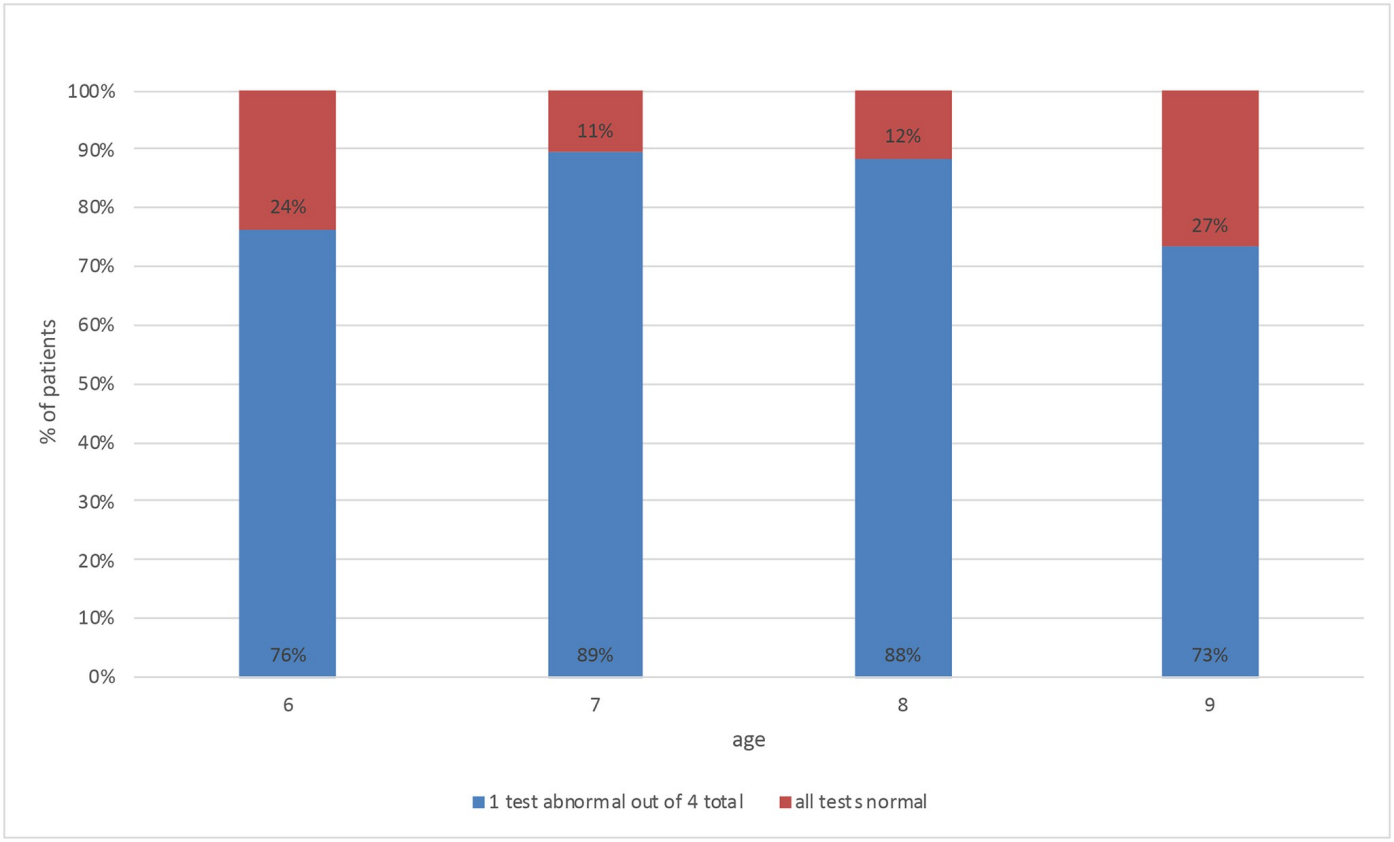
Frequency of abnormal APD test with abnormal results, in group”C”, by age.

The tests with abnormal results were assessed first for all participants by age. In group”C” 82% of the participants obtained abnormal results in one test, the results of the remaining participants were normal in all tests. In group”C” no statistically significant differences were detected among different age groups, with the regard to the number of tests with the results outside the normative range, which was confirmed with a Kruskal-Wallis test (p<0.05). Distribution of the number of abnormal test results in group”C” by age is shown in Fig 4.

**Fig 5:**
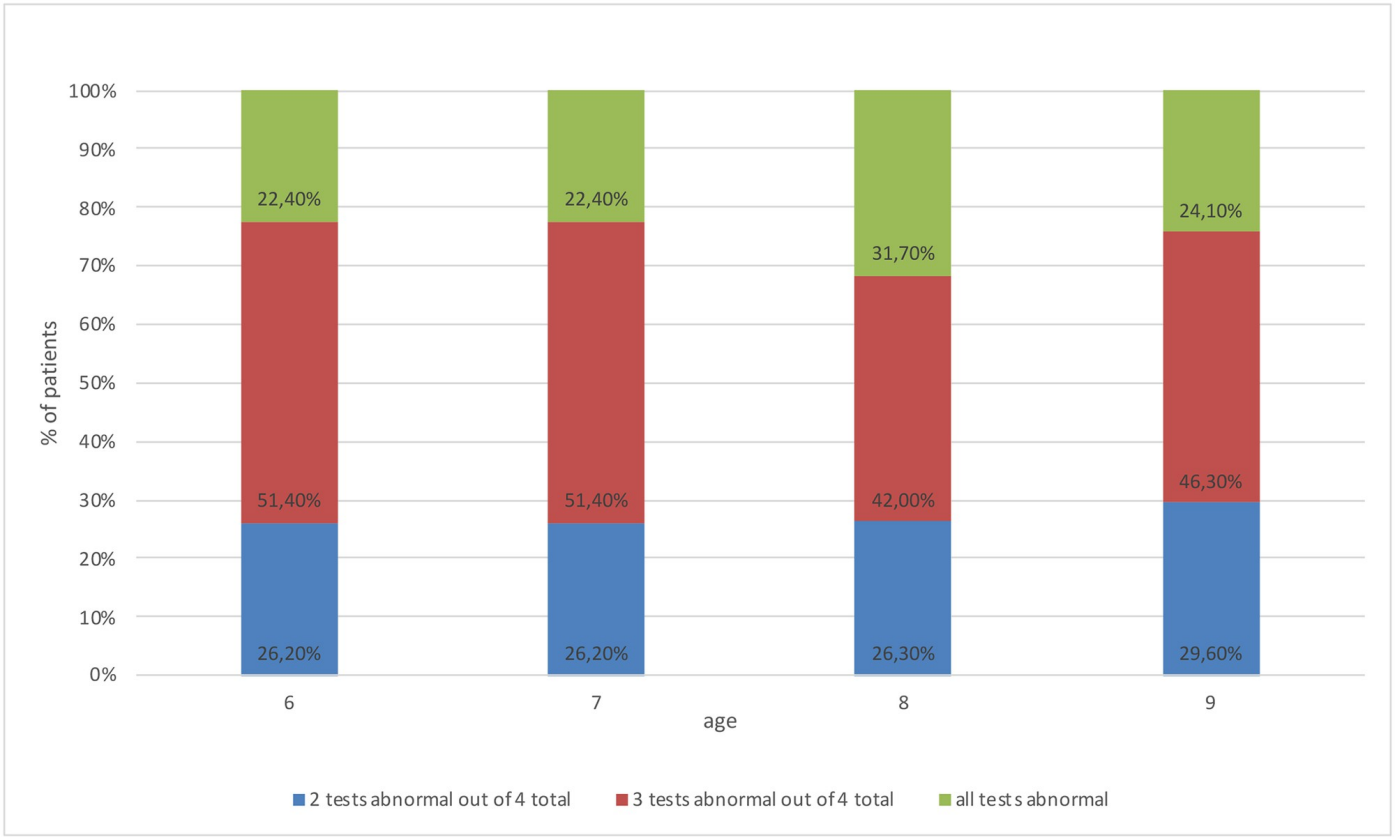
Frequency of abnormal APD test results, by age, in group”S”.

In the”S” group, participants most frequently obtained abnormal results in three tests in each age group, as shown in Fig 5.

It was found that the number of tests with the results below norm and their percentage distribution differ among age groups in group”S” (the test was conducted by the Kruskal-Wallis rank test, the result was obtained at the significance level of p = 0.0094). There is a statistically significant difference between the groups of 7- and 8-year-olds. The results of the post hoc test (a multiple comparison test of mean ranks for all samples) for group”S” are shown in Table 3. The results were considered statistically significant at p<0.05.

**Table 3:** The post hoc analysis of differences among age groups, in terms of the number of tests with abnormal results, for group”S”.

Age (years)	P value for multiple comparisons (two-sided); Independent variable: age Kruskal-Wallis test: H (3, n = 880) = 11.48070 p = 0.0094			
	6 R:448.24	7 R:405.39	8 R:476.07	9 R:441.77
**6**		0.380383	1.000000	1.000000
**7**	0.380383		**0.011538**	0.882863
**8**	1.000000	**0.011538**		1.000000
**9**	1.000000	0.882863	1.000000	

**Fig 6:**
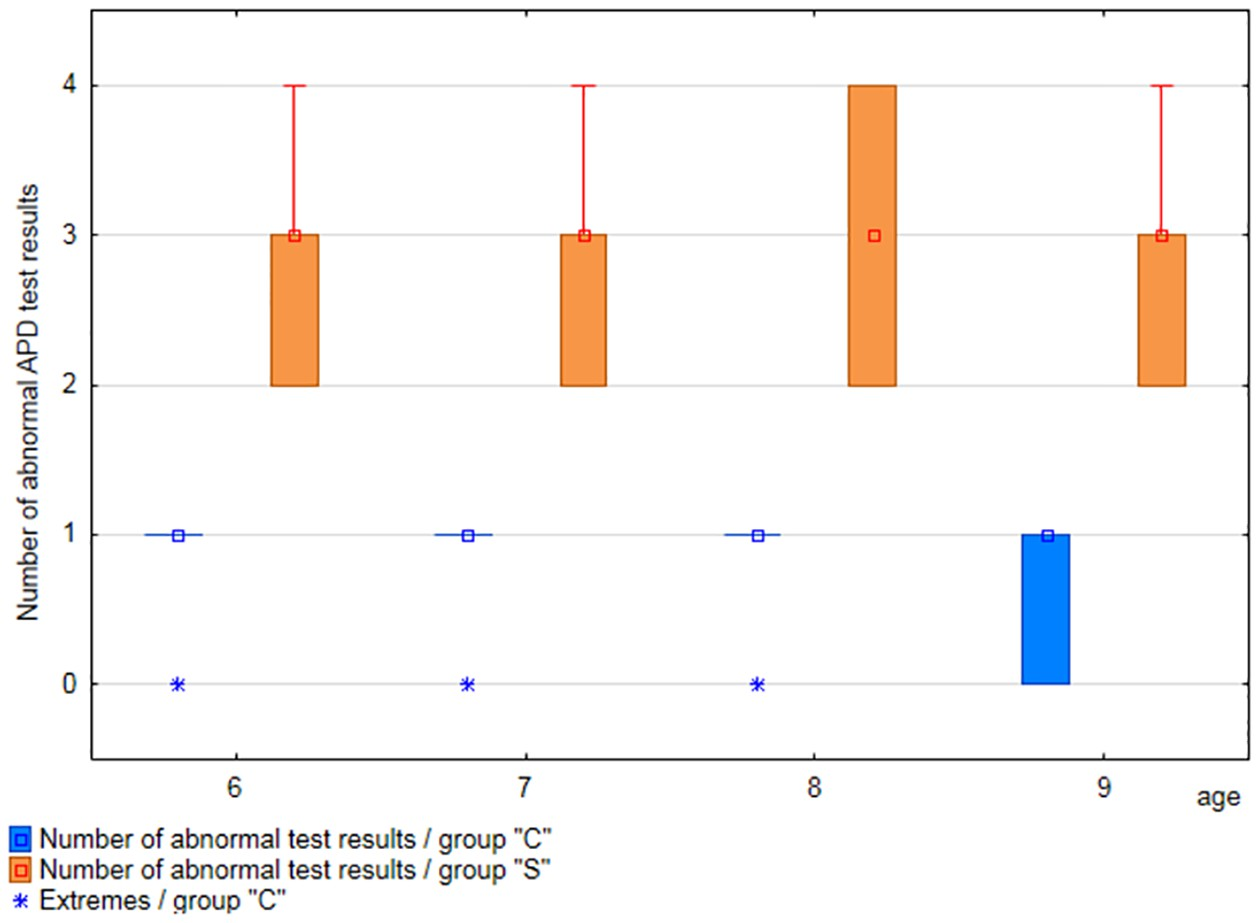
Comparison of the number of abnormal APD test with abnormal results in each study group, by age.

All 8-year-old children (100%) from group "S" obtained abnormal results in two, three or four tests, while 75% of children of other age groups obtained abnormal results in max. three tests (Fig 6).

#### 2.2. The qualitative analysis—The nature of dominant deficit: Test results outside the normative range

The qualitative study was followed by an analysis of a number of tests with abnormal results, from the perspective of which tests were failed with the highest frequency (in each study group, for all age groups). Tables with reference values for each age group can be found in the S5 Table.

All participants obtained the most frequently abnormal results in the ASPN-S test (in each age group): respectively 56% of participants from the group”C” and 92% of participants from the group”S”. DDT was the easiest test for the participants, the abnormal results were found the least.

Auditory lateralization deficits, detected with the application of the DDT, changed with age. The result analysis revealed reversed lateralization patterns of auditory deficits among children in both groups, in each age subgroup, respectively. While the group of participants with APD showed an auditory lateralization deficit in the left ear, the deficit in the right ear dominated in the “C” group. We described the observed phenomenon as”an reversed lateralization pattern of the dichotic listening deficit”.

**Fig 7:**
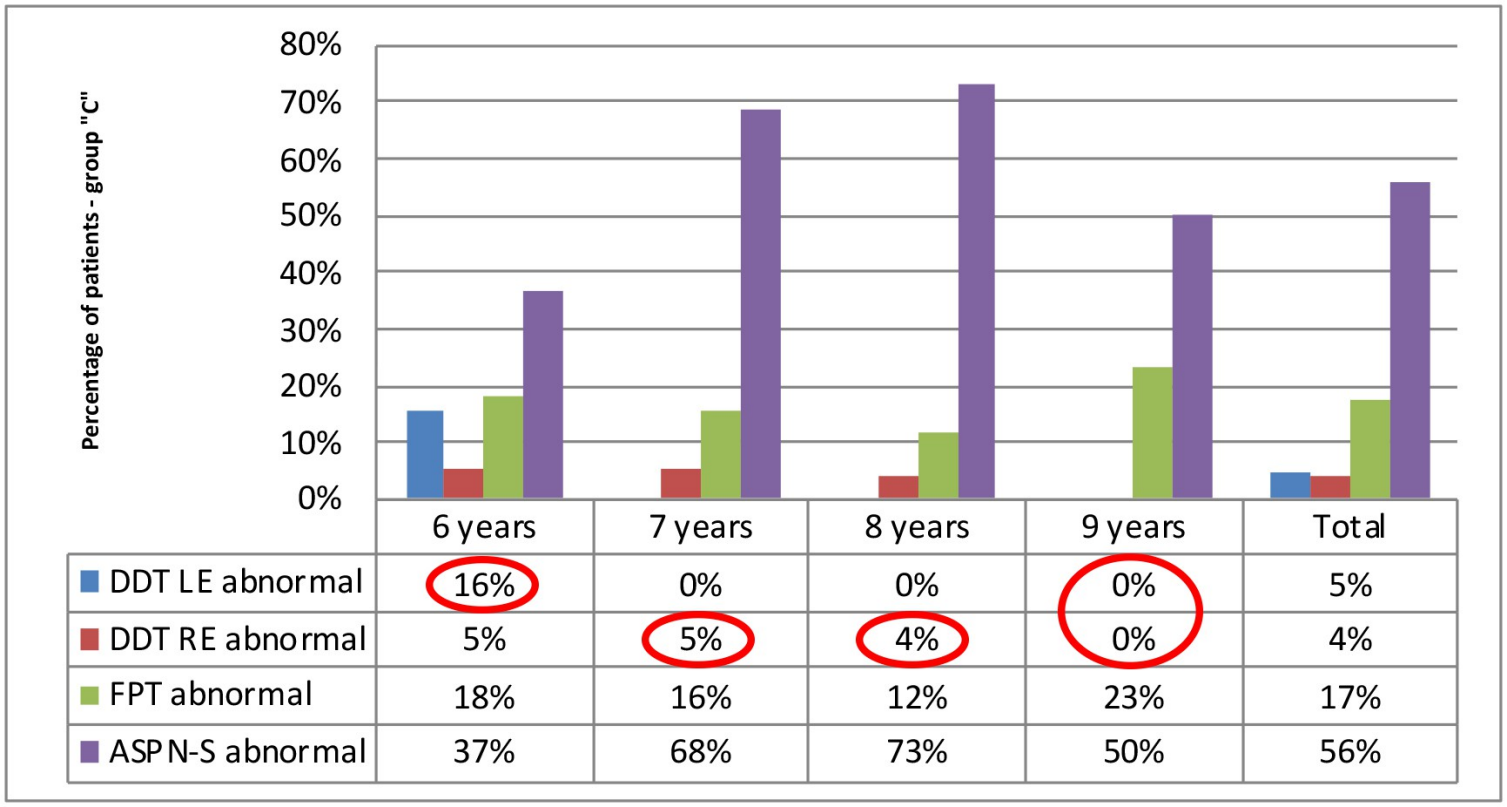
Percentage of abnormal APD test results, by age, in group”C”.

**Fig 8:**
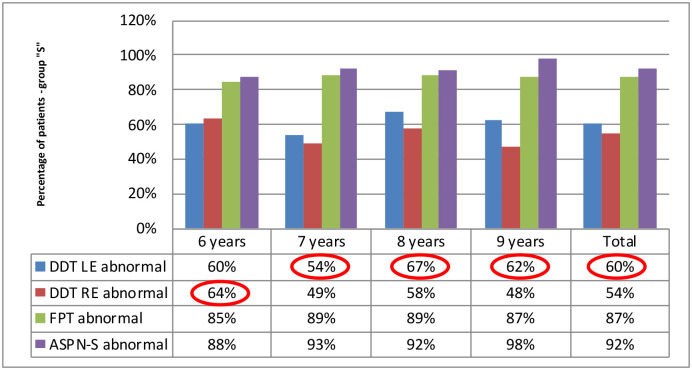
Percentage of abnormal APD test results, by age, in group”S”.

In both study groups, the results of the DDT test obtained by 6-year-olds differed from those of older children. The lateralization of auditory deficits in 6-year-olds was constantly observed in the contralateral ear compared to older children (within a study group). In the”C” group, 6-year-olds obtained abnormal results more frequently for the left ear than for the right ear, while in group”S” they obtained abnormal results more frequently for the right ear than for the left. Right-sided deficit predominated in group”C” in children older than 7, while the left-sided deficit in interaural integration predominated in group”S”. Moreover, in DDT, no deficits in the left ear in children aged over 7 years were found in group”C”. In 9-year-olds, DDT results for both ears were always normal. The detailed distribution of the number of tests with abnormal results (below the age standard), in all tests, by age, is shown in Figs 7 and 8. More frequent results below norm for DDT are circled in red.

A significantly higher frequency of normal DDT results for both ears was found in group”C” in comparison with group”S”. Detailed distribution of results, by age, in both groups is shown in Table 4.

**Table 4:** Percentage of normal results obtained simultaneously for DDT RE and DDT LE, in both study groups, by age.

Normal results of DDT RE and DDT LE, both groups					
Age (years)	6	7	8	9	Total
Groups					
**Group”S”**	8%	24%	14%	20%	**17%**
**Group”C”**	**79%**	**95%**	**96%**	**100%**	**92%**

### 3. The analysis of test result relationships for both examined groups: DDT vs. FPT and DDT vs. ASPN-S test

In the next step, the relationships were assessed for the DDT RE and LE test results and FPT and ASPN-S test results. The Cochran’s Q test was used for dependent samples.

#### 3.1. Relationships between DDT results and ASPN-S test and FPT results in the”C” group

The participants in group”C” who obtained normal results of RE and LE DDT results were more likely to obtain abnormal results in the ASPN-S test (61% of participants) than in FPT (19% of participants) in this group. The detailed result distribution by age is shown collectively in Table 5.

**Table 5:** Percentage of abnormal FPT and ASPN-S test results with normal DDT RE and DDT LE results, in group”C”, by age.

Normal results of DDT RE and DDT LE, group”C”					
Age (years)	6	7	8	9	Total
Tests					
**FPT** **abnormal results**	23%	17%	12%	23%	**19%**
**ASPN-S** **abnormal results**	**47%**	**72%**	**76%**	**50%**	**61%**

Group”C” includes participants with max. one abnormal result in the APD test. Therefore, in this group of participants, it was only possible to analyze either the abnormal FPT test results or abnormal ASPN-S test results with normal DDT results for both ears.

#### 3.2. Relationships between DDT results and ASPN-S test and FPT results in group”S”

In group”S”, when DDT RE and LE results were normal, FPT and ASPN-S test results were always abnormal, and the frequency of obtaining such results by participants was previously shown in Table 4. Therefore, it was only possible to analyze the isolated deficits in FPT and ASPN-S test results for participants with abnormal DDT results for both ears. It was observed that participants were more likely to obtain abnormal results in the ASPN-S test (91%) than in the FPT test (85%), but the disproportion in the frequency of abnormal results in these tests was much lower than in group”C”. Result distribution by age is shown in Table 6.

**Table 6:** Percentage of abnormal FPT and ASPN-S results with abnormal DDT RE and LE results, in group”S”, by age.

Abnormal results of DDT RE and DDT LE, group”S”					
Age (years)	6	7	8	9	Total
Tests					
**FPT** **abnormal results**	83%	82%	89%	84%	**85%**
**ASPN-S** **abnormal results**	**86%**	**93%**	**91%**	**96%**	**91%**

#### 3.3. The influence of lateralization of a dichotic listening deficit on ASPN-S test and FPT results in group”S”

Next, it was assessed what ASPN-S test and FPT results in group”S” were obtained with abnormal DDT results, for a single ear affected. Regardless of lateralization deficits for the function tested with DDT, participants were more likely to obtain abnormal results in ASPN-S test, except for a group of 7-year-olds who in case of deficits found in the right ear were slightly more likely to obtain abnormal results in FPT. Detailed results are shown in Tables 7 and 8.

**Table 7:** Percentage of abnormal FPT and ASPN-S test results with a normal result of the DDT RE test and an abnormal result of DDT LE, group”S”.

Normal results of DDT RE, group”S”					
Age (years)	6	7	8	9	Total
Tests					
**FPT** **abnormal results**	**85%**	86%	87%	92%	**87%**
**ASPN-S** **abnormal results**	**85%**	**91%**	**89%**	**100%**	**91%**

**Table 8:** Percentage of abnormal FPT and ASPN-S test results with a normal result of the DDT LE test and an abnormal result of DDT RE, group”S”.

Normal results of DDT LE, group”S”					
Age (years)	6	7	8	9	Total
Tests					
**FPT** **abnormal results**	82%	**87%**	84%	68%	**82%**
**ASPN-S** **abnormal results**	**90%**	85%	**91%**	**93%**	**89%**

It was not possible to conduct an analogous analysis for the participants from group”C” as they obtained max. one abnormal result in the entire battery of tests.

### 4. The analysis of the profile of auditory function disorders in both analyzed groups, for all age groups

#### 4.1. Assessment of the dynamics of auditory function development in the control group

To trace the dynamics of auditory function development in the healthy population, the test results obtained in individual age groups were compared. Since the Kruskal-Wallis rank-sum test revealed statistically significant differences (for p<0.05) between age groups in terms of the results of all tests assessing auditory functions, a post hoc analysis was performed. The results are presented collectively in Table 9.

**Table 9:** Analysis of differences between age groups for the APD test results in group”C”, with the application of the post hoc (multiple comparisons test of mean ranks for all samples) tests.

Tests	DDT RE	DDT LE	FPT	ASPN-S
**Kruskal–Wallis test**	p = 0.0001	p = 0.0000	p = 0.0065	p = 0.0213
**Differences between age groups**	**6**- and **8**-year-olds (p = 0.006891)	**6**- and **8**-year-olds (p = 0.000336)	**6**- and **8**-year-olds (p = 0.019070)	**6**- and **9**-year-olds (p = 0.031044)
	**6**- and **9**-year-olds (p = 0.000978)	**6**- and **9**-year-olds (p = 0.000001)	**6**- and **9**-year-olds (p = 0.033153)	
	**7**- and **9**-year-olds (p = 0.018518)	**7**- and **9**-year-olds (p = 0.007427)		

It was observed that in terms of the DDT RE and LE tests, the results obtained by children with a one-year age difference were not statistically significant (for p<0.05). On the other hand, a statistically significant difference is visible among the groups with at least two-year age difference (6- and 8-year-olds, 7- and 9-year-olds, and 6- and 9-year-olds). In case of the FPT test, statistically significant differences were observed between the groups of 6- and 8-year-olds and 6- and 9-year-olds. The ASPN-S test, on the other hand, revealed statistically significant differences only between groups of 6- and 9-year-olds.

#### 4.2. Analysis of the APD test results, with regard to age standards, in both study groups

**Fig 9:**
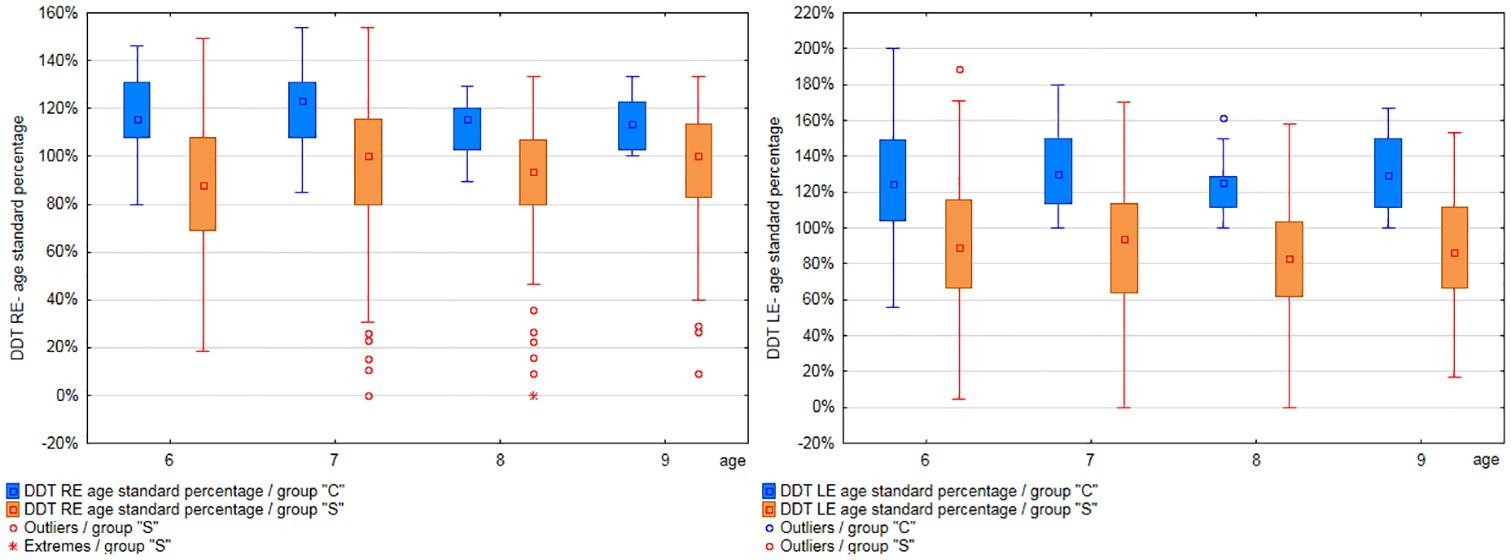
Results for DDT RE and LE (age standard percentage), in 6-, 7-, 8, and 9-year-olds, in groups”S” and”C”.

**Fig 10:**
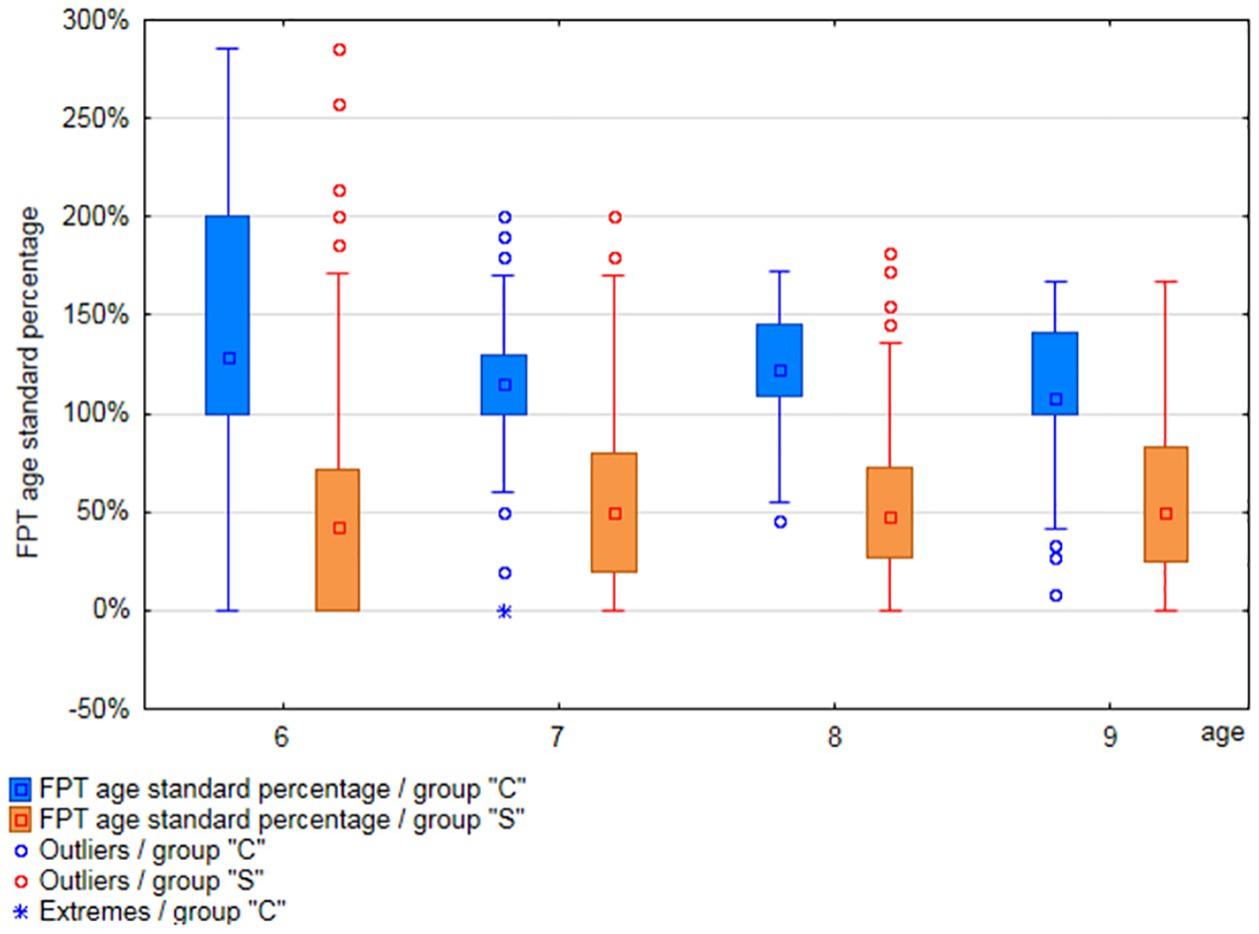
Results of the FPT test (age standard percentage), in 6-, 7-, 8-, and 9-year-olds, in groups”S” and”C”.

The results obtained by each participant were divided by the normative value relevant for the age of the tested person. This allowed to present the results as absolute values (as a percentage) and to compare the results of participants of different ages. The distribution of APD test results presented as a percentage of age standard, in each age group of both study groups, is presented in Figs 9 and 10.

The FPT results, presented as the age standard percentage, in group”S” are significantly lower than in DDT RE and LE, in each age group. In none of the age groups did the median FPT result exceed 50% of the age standard. At the same time, in the group of 6-year-olds, 25% of participants obtained the result of 0% of the age standard.

In case of group”C”, the disproportion was not as significant between the percentages of the standards for FPT, DDT RE and LE. In addition, in the group of 6-year-olds, only 25% of participants obtained results below 100% of the age standard in the FPT test, with the median at the level of 128% of the age standard. The results of all tests (medians) in this group are at a similar level in all age groups.

Due to the difficulties associated with presenting the results of the ASPN-S test as a percentage of the standard, the test results for understanding speech in noise were not analyzed from this perspective.

The results presented as a percentage of standard for a given age were used for the comparison, and the Kruskal-Wallis test was applied. There were statistically significant differences (at the level of p<0.05) between all APD test results in the examined age groups in group "S". Subsequently, the post hoc analysis was performed, the results of which are presented collectively in Table 10.

**Table 10:** Post hoc analysis of differences among age groups, in the percentage of the standard, for each age group, in group”S”.

Tests	DDT RE	DDT LE	FPT
**Significance level**	p = 0.0000	p = 0.0515	p = 0.0179
**Differences between age groups**	**6**- and **7**-year-olds (p = 0.000146)	**7**- and **8**-year-olds (p = 0.039999)	**6**- and **8**-year-olds (p = 0.038093)
	**6**- and **9**-year-olds (p = 0.003951)		**6**- and **9**-year-olds (p = 0.041933)
	**7**- and **8**-year-olds (p = 0.027981)		

Statistically significant differences were found primarily in the DDT RE results (among three groups), but also in the FPT (two groups) and DDT LE (one group). In addition, among six differing groups, four differences concerned 6-year-olds.

Given the obtained results of the post hoc analysis, we compared medians of the percentage of the standard of APD results, across the groups of group”S” (Table 11). The lowest median for the percentage of DDT RE test standard was observed in the group of 6-year-olds, while the lowest median for the DDT LE results was observed in the group of 8-year-olds, and for the results of the FPT test–again in the group of 6-year-olds.

**Table 11:** Comparison of percentage medians of standard APD results, in all age groups, in group”S”.

Tests	DDT RE	DDT LE	FPT
Ages (years)			
**6**	**0.876** *	0.888	**0.428** *
**7**	1.000	0.940	0.500
**8**	0.933	**0.833** *	0.472
**9**	1.000	0.855	0.500

* lowest median

Comparison of APD test results presented as a percentage of the age standard for the participants from group”C”, conducted with the use of the Kruskal-Wallis tests, showed no statistically significant differences among the age groups. Detailed analysis of the level of medians is presented in Table 12.

**Table 12:** Comparison of percentage medians of standard APD results, for all age groups, in group”C”.

Tests	DDT RE	DDT LE	FPT
Ages (years)			
**6**	1.15	1.24	1.28
**7**	1.23	1.30	1.15
**8**	1.15	1.25	1.23
**9**	**1.13** *	**1.13** *	**1.08** *

* lowest median

Analysis of the relationship between the FPT test results and the level of phonemic hearing and auditory memory, in relation to age, is the subject of our current research.

#### 4.3. The analysis of the disproportion between DDT RE and LE results and the developmental dynamics of dichotic listening: A comparison of study groups

The Friedman ANOVA test for dependent samples was used for calculating the mean result obtained by participants of all ages (separately for each age group) for DDT RE and LE. Statistically significant differences were found (for p<0.05) between DDT RE and LE test results. The percentage difference between DDT RE and LE test results was calculated. The summary results are presented in Tables 13 and 14.

**Table 13:** Comparison of mean results for DDT RE and LE (dependent samples) and the percentage difference between the results for group”S”.

Age (years)	6	7	8	9
Tests				
**DDT RE**	57.56	63.26	68.31	72.12
**DDT LE**	40.37	45.46	49.32	53.37
**Difference**	**42.58%**	**39.15%**	**38.50%**	**35.14%**

**Table 14:** Comparison of mean results for DDT RE and LE (dependent samples) and the difference between the results for group”C”.

Age (years)	6	7	8	9
Tests				
**DDT RE**	**76.21**	78.08	84.42	85.63
**DDT LE**	**57.66**	66.76	73.77	78.07
**Difference**	**32.18%**	**16.95%**	**14.44%**	**9.69%**

**Fig 11:**
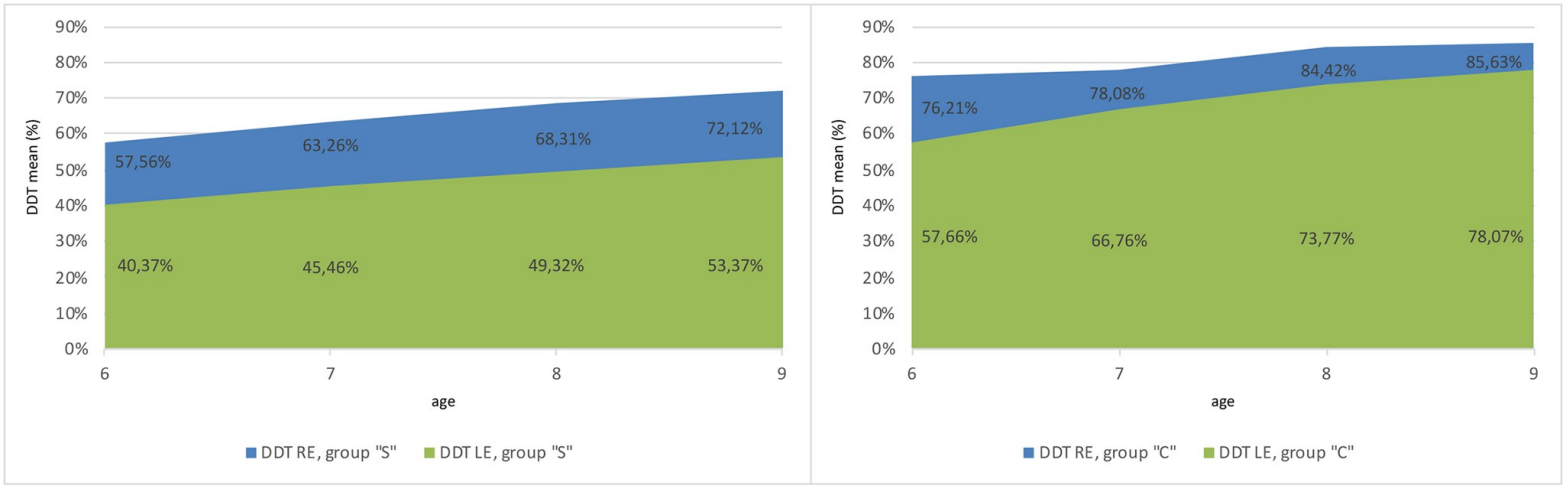
Comparison of mean results for DDT RE and LE (dependent samples) in both groups illustrates right ear dominance in groups”S” and”C”, which decreases with age.

In both analyzed groups, the physiological dominance of the right ear is observed, as acknowledged by other authors [20], which–even though gradually decreasing with age–remains considerably greater in the group of APD children than in the group of children without APD (Fig 11). The difference found in 9-year-old children with APD (35.14%) is greater than that in 6-year-olds without APD (32.18%).

**Fig 12:**
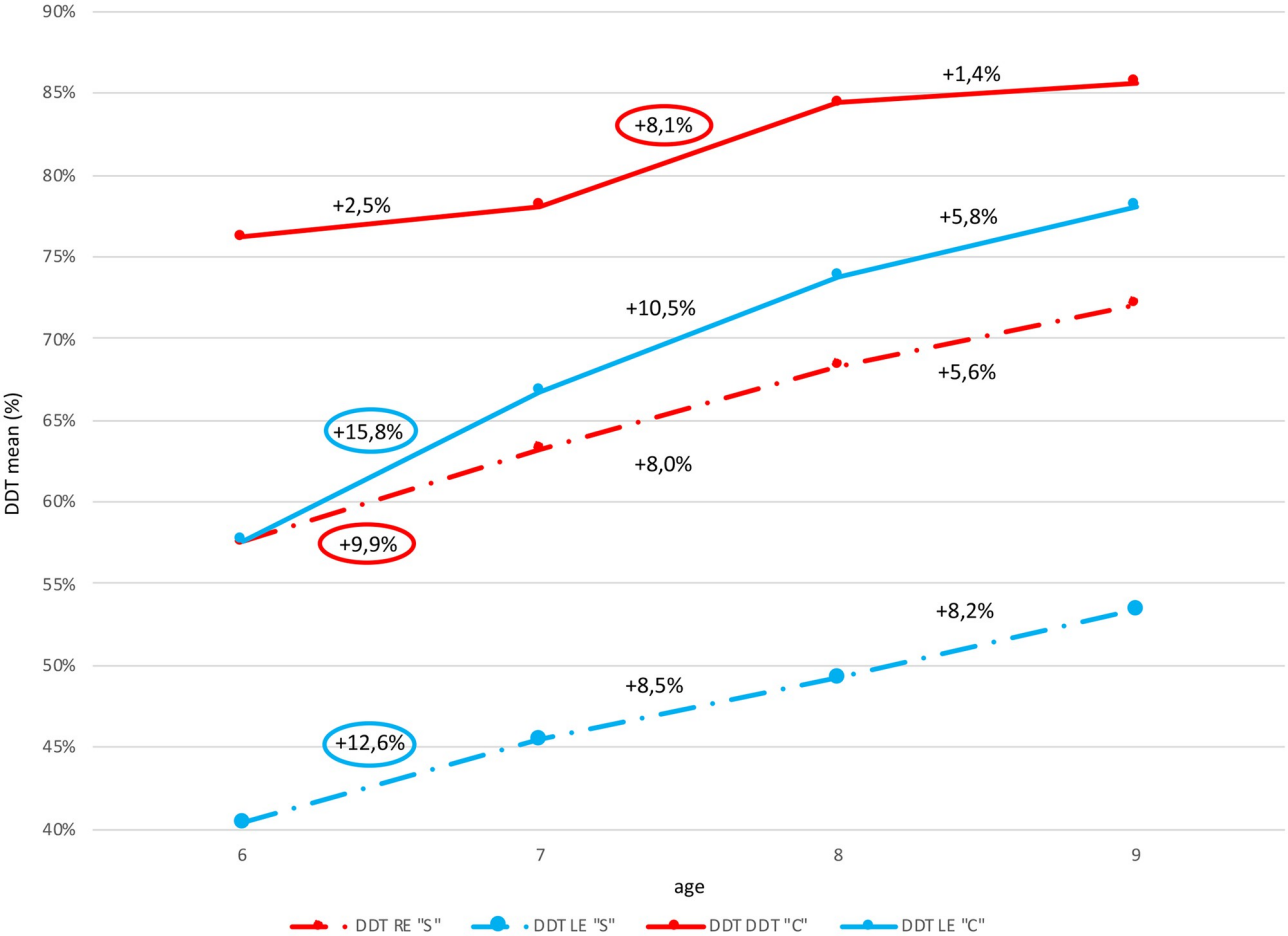
Comparison of dichotic listening development dynamics (mean values of DDT test results for dependent samples), in both study groups, in all age groups.

The developmental dynamics of dichotic listening depending on age was investigated. For this purpose, mean results of DDT tests were compared for both ears across age groups, in both study groups. The results are shown in Fig 12.

The development dynamics of a function measured using DDT depending on age is higher for LE than RE, in both groups. In LE, it decreases with age and is at its peak between the ages of 6 and 7, both in groups”C” and”S”. It represents 15.8% and 12.6%, respectively, as indicated with a blue circle in Fig 12. The development dynamics of RE hearing in group”S” is at its peak between the ages of 6 and 7, and in group”C”–between the ages of 7 and 8, and it represents 9.9% and 8.1%, respectively, as indicated with a red circle in Fig 12.

#### 4.4. Dynamics of the auditory function development: A comparison between the study groups

To assess the dynamics of auditory function development, median results of APD tests obtained by children in both study groups were calculated. Then, the increase in results with age was compared for each diagnosed auditory function in all age groups (year-to-year dynamics is given in brackets). The results are presented in Tables 15 and 16.

**Table 15:** Comparison of median APD results in age groups in group”S”.

Tests	DDT RE	DDT LE	FPT	ASPN-S
Age (years)				
**6**	57*	40*	15*	2.33*
**7**	**65 (+14%)** ^	**47 (+18%)** ^	**25(+67%)** ^	1.33
**8**	70 (+8%)	50 (+6%)	26 (+4%)	1.00
**9**	75** (+7%)	52** (+4%)	30** (+15%)	0.33**

**- highest scores

*—lowest scores

^—highest increase in results

**Table 16:** Comparison of median APD result in age groups in group”C”.

Tests	DDT RE	DDT LE	FPT	ASPN-S
Age (years)				
**6**	75*	56*	45*	-0.5
**7**	80 (+7%)	**65 (+16%)** ^	**57 (+27%)** ^	0*
**8**	**86 (+8%)**^ **	75 (+15%)	67 (+18%) **	0*
**9**	85 (-1%)	77 (+3%) **	65 (-3%)	-1.83 **

**- highest results

*—lowest results

^—highest increase in results

In both study groups, we can observe a development in dichotic listening and frequency discrimination progressing with age. The greatest developmental leap, in terms of the FPT results in both study groups, is observed between the ages of 6 and 7. In addition, in group”S”, there is also (in this age range) a clear increase in the dichotic listening test results in both ears. At the same time, the level of all auditory functions in 9-year-old children with APD–despite the observed normal physiological development–does not exceed the level of functions of 6-year-old children whose auditory functions develop normally.

## Discussion

Auditory processing disorders in children are diagnosed no sooner than at the age of 7, which is argued by an insufficient level of cognitive and linguistic development necessary to carry out meaningful tests in younger children. Despite the prevalence of the disorder in children (up to 10% in the population) [21] and over 60 years of research in the field of APD [22], no diagnostic management standards concerning younger children have been developed to date [23, 24]. The paper presents several reasons for wider and bolder use of the results of auditory function assessment tests in children under the age of 7 in the diagnostic process.

Our results show that the number of APD tests with abnormal results in the group of 6-year-olds is the same as in older children. 6-year-olds with APD most often obtain abnormal results in three out of four conducted tests. In the group of 8-year-olds with APD, 100% of participants obtained results below the standard in two, three or four tests. In other age groups, 75% of examined obtained results above the standard in at least three tests, which signifies that the number of tests with abnormal results is higher in case of 8-year-olds. In the group of participants without APD, the number of tests with abnormal results does not differ across age groups (cf. Figs 4 and 5).

Another aspect confirming the reliability of the results obtained by 6-year-olds is the similar profile of auditory dysfunctions in comparison with older children. The most frequently impaired function is difficulty understanding speech in noise, and the least frequently impaired function is dichotic listening with distracted attention, regardless of the occurrence of APD (cf. Figs 7 and 8).

Lateralization of the DDT deficit does not influence the frequency of abnormal results in the ASPN-S and FPT tests, neither in 6-year-olds nor older children with APD. Patients with left- and right-sided deficit observed in the dichotic test, are more likely to obtain an abnormal result in the ASPN-S test, except for the group of 8-year-old children. Only slightly more often, patients with a right-sided deficit obtained results below the standard in the FPT test (cf. Tables 7 and 8).

The nature of the lateralization of the deficit in the DDT test results depends on the presence of APD (cf. Table 17). The term”reversed lateralization pattern of dichotic listening deficit” has been used to describe the observed phenomenon.

**Table 17:** Reversed lateralization pattern of dichotic listening deficit in DDT depending on the prevalence of APD, by age (fields in red or green indicate results below or within standard, respectively).

		6	7	8	9
**Group”C”**	**DDT LE**	**↓**	**↑**	**↑**	**↑**
	**DDT RE**		**↓**	**↓**	**↑**
**Group”S”**	**DDT LE**		**↓**	**↓**	**↓**
	**DDT RE**	**↓**			

The results of the conducted studies indicate that the development of interaural integration functions in healthy children (between the age of 6 and 9) progresses in intervals of at least two years (cf. Table 9). It indicates that statistically significant developmental leaps can be expected after this time, perhaps also in the assessment of the therapeutic progress in relation to DDT test results for both ears [25]. However, this requires further research which will assess the dynamics of auditory function development during the conducted therapy. Therefore, mere observation (follow-up) of the development of this function at the ages of 6 or 7, without any resolute therapeutic action, seems to be pointless.

In case of the development of the function assessed with the use of the FPT test, the study also revealed statistically significant differences only at two-year intervals for the group of 6-year-olds (cf. Table 9). It means that in children between the ages of 6 and 9, the ability to differentiate sounds in three-element sequences develops at a slower rate than within a period of one–or even two–years (no statistically significant difference was observed in case of 7-year-olds compared to children who were one and two years older, and in the group of 8-year-olds–compared to children who were one year older) [25]. Further studies which take into account the results of older age groups are recommended.

The dynamics of the obtained results is even slower when it comes to the ability to understand distorted speech. Statistically significant differences were observed only in children with at least three-year difference (cf. Table 9). Due to the development rate of that auditory function and its significant impact on a child’s everyday functioning, a lower ASPN-S result, together with the clinical symptoms reported by parents and after excluding other causes, should be an absolute indication for intensive training aimed at improving the understanding of deformed speech, or for equipping the youngest children with the FM system [25–28].

The results obtained in the FPT tests by our participants from the "S" group are twice lower than those obtained by healthy children (cf. Tables 11 and 12). However, these disproportions are at the same level (as recalculated into percentage, age-unrelated, values). The degree of difficulty of this test often raises questions about its reliability in younger children. However, the results of this study showed that the frequency and severity of the disorder are similar in participants ages 6–9 years, in both healthy children and those with APD. At the same time, in the group of children with APD, the results of the FPT in relation to the age norms are significantly lower than in the dichotic tests. That allows the conclusion that the frequency pattern test is the most difficult one for many children with APD. Such a disproportion in results is not observed in the population of children without APD. Significantly lower results in the FPT in terms of age standards, with borderline values of DDT results, may affect therapeutic decisions. Abnormal results in the FPT test, also due to the lower dynamics of the development of this function compared to the results obtained in the DDT test, indicate the need for training this function, regardless of the child’s age. The results obtained in the FPT tests in the diagnostic evaluation of six-year-olds will help improve the determination of therapeutic goals. The test assesses the ability to differentiate between sound frequencies, but also measures the level of short-term auditory memory. If one of those is disordered, it will affect the overall test result. The assessment of verbal auditory memory, consisting of repeating sequences of digits, can be useful when verifying the impact of the memory factor on the final result of the FPT test. Early musical education, which fosters the development of the auditory pathway, can have a positive impact on the development of these skills [29–32].

A clear developmental leap in dichotic listening function with distracted attention (analyses for dependent samples) is observed in children aged between 6 and 7 (cf. Fig 12). Therefore, DDT test performed at the age of 6, and perhaps an elaboration of normative values for 5-year-olds, would enable effective use of the period which is particularly sensitive for auditory pathway development.

In children with auditory processing disorders, despite their normal physiological development, the level of all examined auditory functions at the age of 9 did not exceed the level reached by 6-year-olds in the healthy population (cf. Tables 15 and 16). It means that without therapeutic assistance, children with delayed development of auditory functions are incapable of compensating for developmental deficits in the first years of school (until the age of 9), which is so important for further education. Since the neuroplasticity of the auditory pathway significantly decreases after the age of 7 [33], it is particularly important that therapeutic actions were implemented as soon as possible, and their goals can be better determined with the use of test results assessing higher auditory functions.

Based on these observations, it is recommended that therapeutic decisions were taken as early as possible, also in younger children, with delayed or disturbed development of the auditory portion of the nervous system. While developmental leaps occur after relatively long periods [25], mere observation of a child’s development while waiting for the physiological development of the auditory pathway may turn out to be an unnecessary delay which will result in problems at school and, consequently, worse achievements. Early interventions may help ensure an equitable position at the beginning of education and prevent secondary deficits in the child’s development. It is worth noting that in the analysis conducted in the paper, the worst results in terms of the number of tests with abnormal results were observed in case of 8-year-olds in group”C” (cf. Fig 6). It may stem from a conservative approach to the study and assessment of auditory functions, as well as the introduction of therapy two years earlier (when the current 8-year-olds were aged 6), when potential abnormal test results were approached with caution.

However, developing auditory pathways and still-developing auditory functions should not stand in the way of conducting a risk assessment of auditory processing disorder aimed at early intervention to stimulate the auditory system [34]. Early evaluation should be a commonly applied diagnostic path in children under 7 with auditory perception difficulties and factors which may disturb or delay the auditory functions development [35–37].

The consequences of late diagnosis of auditory processing disorders, i.e. after the beginning of school education, are crucial for the child’s further development and future educational success. Therefore, it is necessary to assess the risk of auditory processing disorders, especially among children from risk groups, and to implement appropriate preventive or therapeutic measures. This is particularly important because the stimulation of auditory and phonological functions through play is not an invasive therapy and can be a part of everyday activities with a parent or a guardian, as well as a key to the child’s future educational successes.

## Supporting information

S1 Table Early clinical symptoms of APD (authors’ own work). (DOCX)

S2 Table Risk factors for APD (authors’ own work). (DOCX)

S3 Table Tests for normality to verify distribution of data. The results of the verification tests of the normality of distribution are summarized in S3 Table. Test value (*p*) below *0*.*05* indicates rejection of the null hypothesis (H0) assuming normal distribution. These values are marked in red in the table.
(DOCX)

S4 Table Dataset. All individual measurements for ASPN-S, DDT LE and RE, FPT tests collected in the study.
(DOCX)

S5 Table Reference values for ASPN-S, DDT LE, DDT RE, FPT tests. (DOCX)

S6 Table **1**. Basic statistics describing the DDT test results for the RE (top) and LE (bottom) in study group”S” and control group”C”, by age group. **2**. Basic statistics describing the FPT test results in study and control groups (”S” and”C” respectively), by age group. **3**. Basic statistics describing the ASPN-S test results in study and control groups (”S” and”C” respectively), by age group.
(DOCX)
