# Supplementary material for: Analysis of the auditory processing skills in 1,012 children aged 6–9 confirms the adequacy of APD testing in 6-year-olds
Source: PLoS One. 2022 Aug 18;17(8):e0272723. doi: 10.1371/journal.pone.0272723 (PMC9387814; doi:10.1371/journal.pone.0272723)
Supplement: S1 Table — (DOCX) [file pone.0272723.s001.docx]

**Table S1. Early clinical symptoms of APD (authors' own work).**

| Symptoms which may indicate APD in children younger than 7 include the following: |
| --- |
| - inconsistent response to sounds or commands, a need to repeat commands, - difficulty in understanding instructions conveyed via the auditory pathway and in understanding complex instructions, - difficulty in understanding distorted speech or speech under adverse acoustic conditions, - better comprehension with the assistance of visual indications, searching for hints  from other sensory modalities, - difficulty in locating the sound source, - difficulties in keeping attention, especially in a group, - difficulties in remembering poems, songs, - confusing similarly sounding words, confusing song lyrics, - difficulties in mastering synthesis and syllable analysis, and phonemic synthesis at an older age, - difficulties in identifying or creating rhymes, - preference for visual play and activities, reluctance to listen to books when read aloud, - looking lost in a group, carrying out commands or joining games with some delay compared to peers, watching what the peers do, - failure to initiate social contacts, social withdrawal, - hypersensitive hearing, avoiding noisy social situations, covering one’s ears with hands or even crying, - reluctance to talk on the phone, - auditory fatigue after being in a noisy environment (e.g. in kindergarten, at birthday parties, fairs), - articulation disorder, inarticulate speech. |
