# Supplementary material for: Analysis of the auditory processing skills in 1,012 children aged 6–9 confirms the adequacy of APD testing in 6-year-olds
Source: PLoS One. 2022 Aug 18;17(8):e0272723. doi: 10.1371/journal.pone.0272723 (PMC9387814; doi:10.1371/journal.pone.0272723)
Supplement: S2 Table — (DOCX) [file pone.0272723.s002.docx]

**Table S2.** **Risk factors for APD (authors' own work).**

| Risk factors for APD: |
| --- |
| - meningitis, - eventful perinatal history, - premature birth, - perinatal hypoxia, - hyperbilirubinemia, - intrauterine infection with Toxoplasma gondii and cytomegalovirus, - auditory deprivation related to conductive hearing loss in the course of recurrent exudative otitis media, - Eustachian tube failure [1], - hypertrophy of the third tonsil, - chronic upper respiratory tract infections, - oral resting breath pattern [2], - allergies. |

**References**

[1] **Paczkowska A, Marcinkowski J.** Istota zaburzeń przetwarzania słuchowego - niedocenianego problemu zdrowotnego, Hygeia Public Health 2013, 48(40): 396-399.

[2] **Senderski A.** Rozpoznawania i postępowanie w zaburzeniach przetwarzania słuchowego u dzieci. Otolaryngologia 2014, 13 (2): 77-81.
