# Supplementary material for: Analysis of the auditory processing skills in 1,012 children aged 6–9 confirms the adequacy of APD testing in 6-year-olds
Source: PLoS One. 2022 Aug 18;17(8):e0272723. doi: 10.1371/journal.pone.0272723 (PMC9387814; doi:10.1371/journal.pone.0272723)
Supplement: S3 Table — The results of the verification tests of the normality of distribution are summarized in S3 Table. Test value (p) below 0.05 indicates rejection of the null hypothesis (H0) assuming normal distribution. These values are marked in red in the table. (DOCX) [file pone.0272723.s003.docx]

**Table S3. Tests for normality to verify distribution of data.**

The results of the verification tests of the normality of distribution are summarized in Table S3. Test value (*p*) below *0.05* indicates rejection of the null hypothesis (H0) assuming normal distribution. These values are marked in red in the table.

| **Group "C": Kolmogorov–Smirnov test and Lilliefors test** | | | | | | | | | | |
| --- | --- | --- | --- | --- | --- | --- | --- | --- | --- | --- |
| **Age** | **Total** | | **Age: 6** | | **Age: 7** | | **Age: 8** | | **Age: 9** | |
| **Test** | **KS** | **LF** | **KS** | **LF** | **KS** | **LF** | **KS** | **LF** | **KS** | **LF** |
| **DDT LE** | NS | <0.05 | NS | NS | NS | <0.10 | NS | NS | NS | <0.10 |
| **DDT RE** | NS | <0.05 | NS | NS | NS | NS | NS | <0.10 | NS | <0.10 |
| **FPT** | NS | <0.01 | NS | <0.10 | <0.10 | <0.01 | NS | <0.20 | <0.15 | <0.01 |
| **ASPN-S** | NS | <0.05 | NS | <0.01 | NS | <0.10 | NS | <0.10 | NS | <0.05 |
| **Group "S": Kolmogorov–Smirnov test and Lilliefors test** | | | | | | | | | | |
| **Age** | **Total** | | **Age: 6** | | **Age: 7** | | **Age: 8** | | **Age: 9** | |
| **Test** | **KS** | **LF** | **KS** | **LF** | **KS** | **LF** | **KS** | **LF** | **KS** | **LF** |
| **DDT LE** | <0.20 | <0.01 | NS | <0.05 | NS | <0.01 | NS | <0.05 | NS | NS |
| **DDT RE** | NS | NS | NS | NS | <0.05 | <0.01 | <0.10 | <0.01 | <0.05 | <0.01 |
| **FPT** | <0.01 | <0.01 | <0.01 | <0.01 | <0.01 | <0.01 | <0.01 | <0.01 | <0.05 | <0.01 |
| **ASPN-S** | <0.01 | <0.01 | <0.01 | <0.01 | <0.05 | <0.01 | <0.10 | <0.01 | <0.01 | <0.01 |
| **Group "C" and "S": Shapiro-Wilk test** | | | | | | | | | | |
| **Age** | **Total** | | **Age: 6** | | **Age: 7** | | **Age: 8** | | **Age: 9** | |
| **Test** | **Group "C"** | **Group "S"** | **Group "C"** | **Group "S"** | **Group "C"** | **Group "S"** | **Group "C"** | **Group "S"** | **Group "C"** | **Group "S"** |
| **DDT LE** | 0.15 | 0.00 | 0.43 | 0.43 | 0.07 | 0.03 | 0.33 | 0.09 | 0.09 | 0.14 |
| **DDT RE** | 0.08 | 0.00 | 0.55 | 0.29 | 0.64 | 0.00 | 0.20 | 0.00 | 0.02 | 0.00 |
| **FPT** | 0.00 | 0.00 | 0.00 | 0.00 | 0.30 | 0.00 | 0.07 | 0.00 | 0.18 | 0.00 |
| **ASPN-S** | 0.02 | 0.00 | 0.26 | 0.00 | 0.01 | 0.00 | 0.19 | 0.00 | 0.02 | 0.00 |

**KS-** Kolmogorov–Smirnov test, **LF-** Lilliefors test, **NS -** Not Statistically Significant
