# Supplementary material for: Analysis of the auditory processing skills in 1,012 children aged 6–9 confirms the adequacy of APD testing in 6-year-olds
Source: PLoS One. 2022 Aug 18;17(8):e0272723. doi: 10.1371/journal.pone.0272723 (PMC9387814; doi:10.1371/journal.pone.0272723)
Supplement: S4 Table — All individual measurements for ASPN-S, DDT LE and RE, FPT tests collected in the study. (DOCX) [file pone.0272723.s004.docx]

**Table S4. Dataset. All individual measurements for ASPN-S, DDT LE and RE, FPT tests collected in the study** (XLSX file, DOI: [dx.doi.org/10.17504/protocols.io.b5w6q7he](https://dx.doi.org/10.17504/protocols.io.b5w6q7he)**).**
