# Supplementary material for: Analysis of the auditory processing skills in 1,012 children aged 6–9 confirms the adequacy of APD testing in 6-year-olds
Source: PLoS One. 2022 Aug 18;17(8):e0272723. doi: 10.1371/journal.pone.0272723 (PMC9387814; doi:10.1371/journal.pone.0272723)
Supplement: S5 Table — (DOCX) [file pone.0272723.s005.docx]

**Table S5. Reference values for ASPN-S, DDT LE, DDT RE, FPT tests.**

| **Age** | **ASPN-S** | **DDT LE** | **DDT RE** | **FPT** |
| --- | --- | --- | --- | --- |
| **6** | SNR 0 dB | 45% | 65% | 35% |
| **7** | SNR -1 dB | 50% | 65% | 50% |
| **8** | SNR -2 dB | 60% | 75% | 55% |
| **9** | SNR -2 dB | 60% | 75% | 60% |

The interpretation of the results of tests:

- ASPN-S: the value was considered to be normative when the result obtained was lower than the reference value for the age group

- DDT LE, DDT RE, FPT: the value was considered to be normative when the result obtained was higher than the reference value for the age group
