# Supplementary material for: Analysis of the auditory processing skills in 1,012 children aged 6–9 confirms the adequacy of APD testing in 6-year-olds
Source: PLoS One. 2022 Aug 18;17(8):e0272723. doi: 10.1371/journal.pone.0272723 (PMC9387814; doi:10.1371/journal.pone.0272723)
Supplement: S6 Table — 1. Basic statistics describing the DDT test results for the RE (top) and LE (bottom) in study group”S” and control group”C”, by age group. 2. Basic statistics describing the FPT test results in study and control groups (”S” and”C” respectively), by age group. 3. Basic statistics describing the ASPN-S test results in study and control groups (”S” and”C” respectively), by age group. (DOCX) [file pone.0272723.s006.docx]

**Table S6.1. Basic statistics describing the DDT test results for the RE (top) and LE (bottom) in study group ”S” and control group ”C”, by age group.**

| **DDT RE** | | | | | | | | | | |
| --- | --- | --- | --- | --- | --- | --- | --- | --- | --- | --- |
|  | **Number of examined** | | **Mean (SD)** | | **Median** | | **Min** | | **Max** | |
| **Group**  **Age** | **”C”** | **”S”** | **”C”** | **”S”** | **”C”** | **”S”** | **”C”** | **”S”** | **”C”** | **”S”** |
| **6** | 38 | 214 | 76.21 (9.48) | 57.36 (17.25) | 75 | 57 | 52 | 12 | 95 | 97 |
| **7** | 38 | 280 | 78.08 (10.73) | 63.26 (18.14) | 80 | 65 | 55 | 0 | 100 | 100 |
| **8** | 26 | 224 | 84.42 (7.9) | 68.31 (17.44) | 86,5 | 70 | 67 | 0 | 97 | 100 |
| **9** | 30 | 162 | 85.63 (8.24) | 72.12 (16.04) | 85 | 75 | 75 | 7 | 100 | 100 |
| **Total** | 132 | 880 | 80.5 (10) | 64.7 (18.1) | 80 | 67 | 52 | 0 | 100 | 100 |
| **DDT LE** | | | | | | | | | | |
|  | **N** | | **Mean (SD)** | | **Median** | | **Min** | | **Max** | |
| **Group**  **Age** | **”C”** | **”S”** | **”C”** | **”S”** | **”C”** | **”S”** | **”C”** | **”S”** | **”C”** | **”S”** |
| **6** | 38 | 214 | 57.66 (16.11) | 40.37 (16.37) | 56 | 40 | 25 | 2 | 90 | 85 |
| **7** | 38 | 280 | 66.76 (11.38) | 45.46 (18.37) | 65 | 47 | 50 | 0 | 90 | 85 |
| **8** | 26 | 224 | 73.77 (9.05) | 49.32 (19.03) | 75 | 50 | 60 | 0 | 97 | 95 |
| **9** | 30 | 162 | 78.07 (12.14) | 53.37 (18.69) | 77,5 | 52 | 60 | 10 | 100 | 92 |
| **Total** | 132 | 880 | 68.1 (14.8) | 46.7 (18.7) | 69 | 47 | 25 | 0 | 100 | 95 |

**Table S6.2. Basic statistics describing the FPT test results in study and control groups (”S” and ”C” respectively), by age group.**

| **FPT** | | | | | | | | | | |
| --- | --- | --- | --- | --- | --- | --- | --- | --- | --- | --- |
|  | **N** | | **Mean (SD)** | | **Median** | | **Min** | | **Max** | |
| **Group**  **Age** | **”C”** | **”S”** | **”C”** | **”S”** | **”C”** | **”S”** | **”C”** | **”S”** | **”C”** | **”S”** |
| **6** | 38 | 214 | 50.66 (24.64) | 18.20 (21) | 45 | 15 | 0 | 0 | 0 | 100 |
| **7** | 38 | 280 | 57.24 (20.49) | 25.81 (19.84) | 57,5 | 25 | 0 | 0 | 100 | 100 |
| **8** | 26 | 224 | 66.35 (16.82) | 29.62 (20.56) | 67,5 | 26 | 25 | 0 | 95 | 100 |
| **9** | 30 | 162 | 64.03 (25.71) | 33.08 (22.9) | 65 | 30 | 5 | 0 | 100 | 100 |
| **Total** | 132 | 880 | 58.7 (23) | 26.3 (21.5) | 60 | 23 | 0 | 0 | 100 | 100 |

**Table S6.3. Basic statistics describing the ASPN-S test results in study and control groups (”S” and ”C” respectively), by age group.**

| **ASPN-S** | | | | | | | | | | |
| --- | --- | --- | --- | --- | --- | --- | --- | --- | --- | --- |
|  | **N** | | **Mean (SD)** | | **Median** | | **Min** | | **Max** | |
| **Group**  **Age** | **”C”** | **”S”** | **”C”** | **”S”** | **”C”** | **”S”** | **”C”** | **”S”** | **”C”** | **”S”** |
| **6** | 38 | 214 | 0.15  (2.07) | 2.92 (2.6) | -0.5 | 2.33 | -2.33 | -3.67 | 7 | 9 |
| **7** | 38 | 280 | -0.33 (1.18) | 1.67 (2.12) | 0 | 1.33 | -2.67 | -3 | 2 | 9 |
| **8** | 26 | 224 | -0.1 (1.81) | 1.19 (2.4) | 0 | 1 | -3 | -4 | 2.67 | 8.33 |
| **9** | 30 | 162 | -1.23 (1.8) | 0.84 (2.1) | -1.83 | 0.33 | -4.33 | -3 | 3.67 | 9 |
| **Total** | 132 | 880 | -0.4 (1.8) | 1.7 (2.4) | -0.7 | 1.3 | -4.3 | -4 | 7 | 9 |
